# Effect of empathy training on the empathy level of healthcare providers in Ethiopia: a cluster randomized controlled trial

**DOI:** 10.3389/fpsyg.2023.1091605

**Published:** 2023-05-22

**Authors:** Bekana Fekecha Hurissa, Zewdie Birhanu Koricha, Lelisa Sena Dadi

**Affiliations:** ^1^School of Midwifery, Institute of Health, Jimma University, Jimma, Ethiopia; ^2^Department of Health, Behavior, and Society, Faculty of Public Health, Jimma University, Jimma, Ethiopia; ^3^Department of Epidemiology, Faculty of Public Health, Jimma University, Jimma, Ethiopia

**Keywords:** effect, Ethiopia, empathy training, empathy level, healthcare providers’ empathy

## Abstract

**Objective:**

Empathy has deteriorated throughout clinical training and medical practice, and little is known about the effect of empathy training on the empathy level of healthcare providers. To address this gap, we assessed the effect of empathy training on the empathy level of healthcare providers in Ethiopia.

**Design:**

A cluster randomized controlled trial study design was conducted from 20 December 2021 to 20 March 2022. The empathy training intervention was conducted for three consecutive days.

**Setting:**

The study was conducted in five fistula treatment centers in Ethiopia.

**Participants:**

The participants were all randomly selected healthcare providers.

**Main outcome measures:**

Total mean score, percentage changes, and Cohen’s effect size were computed. A linear mixed effects model and independent *t*-test were used for data analysis.

**Results:**

A majority of the study participants were nurses in the profession, married, and first-degree holders. There was no statistically significant difference in the baseline empathy score of the intervention arm across their socio-demographic features. At the baseline, the mean empathy scores of the control and intervention arms were 102.10 ± 15.38 and 101.13 ± 17.67, respectively. The effect of empathy training on the total mean score changes of empathy of the intervention arm compared to the control arm at each follow-up time had a statistically significant difference. After a week, a month, and three months of post-intervention, the total mean empathy scores between the intervention and control arms were as follows: (intervention 112.65 ± 18.99, control 102.85 ± 15.65, *d* = 0.55, *p* = 0.03); (intervention 109.01 ± 17.79, control 100.52 ± 12.57, d = 0.53, *p* = 0.034); and (intervention 106.28 ± 16.24, control 96.58 ± 14.69, *d* = 0.60, *p* = 0.016) with the overall percentage changes of 11, 8, and 5% from the baseline scores, respectively.

**Conclusion:**

In this trial, the empathy training intervention was found to have more than a medium effect size. However, over the follow-up intervals, there was a decreasing trend in the total mean empathy scores of healthcare providers; suggesting that there should be continued empathy training and integration of it into educational and training curriculums to enhance and sustain the empathy of healthcare providers.

**Clinical Trial Registration**: Pan African Clinical Trial Registry: http://www.edctp.org/panafrican-clinical-trials-registry or https://pactr.samrc.ac.za, PACTR202112564898934.

## Introduction

Within the fields of both medicine and allied healthcare, approaching patient care with a heightened level of empathetic behavior has been shown to achieve greater positive outcomes (Williams et al., 2015a). The term empathy is derived from the Greek word *empatheia*, which means appreciation of another person’s feelings. This definition of empathy is the first description of the significance of empathy in the relationship between a clinician and a patient for facilitating diagnostic outcomes (Clark, 2010). Empathy is considered the basis of effective communication and one of the most important skills to be developed by human beings; the ability to put oneself in the place of other people so that a person can visualize and feel the experiences of other(s) from the same perspective; and a fundamental attitude for the physical and mental wellbeing of both the empathizer and the target (Terezam et al., 2017).

Much of the literature supports the belief that both cognitive and emotional empathy approaches are multifaceted and differentiated from sympathy through the identification of others’ feelings while limiting personal involvement and maintaining clinical neutrality (Ward et al., 2012). Cognitive empathy is the ability to understand another person’s feelings, related closely to the theory of mind (Blair, 2005). Lamm suggested that while affective empathy is automatically elicited, manipulation of cognitive elements can modulate affective elements or the cognitive component is the process *via* which affective content is formed (Lamm et al., 2007). Affective empathy is concerned with the experience of emotion elicited by an emotional stimulus. The key feature of empathy is the preponderance of cognitive information processing that distinguishes it from the predominantly emotional mental processing involved in sympathy (Clark, 2010; Singer and Klimecki, 2014). Cognitive empathy leads to personal growth, career satisfaction, and optimal clinical outcomes, whereas affective sympathy can lead to career burnout, compassion fatigue, exhaustion, and vicarious traumatization. Affect and emotion (the prominent ingredients of sympathy) are less amenable to change, whereas cognition and understanding (the prominent ingredients of empathy) can be substantially enhanced by education (Hojat, 2016e).

Empathy in the context of patient care is defined as a predominantly cognitive (rather than an affective or emotional) attribute that involves an understanding (rather than feeling) of the experiences, concerns, and perspectives of the patient, combined with a capacity to communicate this understanding, and an intention to help (Jeffrey, 2016; Hojat, 2016a). The cognition element of empathy includes reasoning and appraisal, which are the basis of clinical judgment (Hojat, 2016c). “Understanding is also a key ingredient of empathetic engagement in the healthcare provider–patient relationship” (Bauchat et al., 2016; Sinclair et al., 2017). A specific feature of understanding in a healthcare provider–patient relationship is the ability to stand in a patient’s shoes (knowing that the shoes belong to someone else), and to view the world from the patient’s perspective without losing sight of one’s role and professional responsibilities (Hojat, 2016e). Communication of understanding is also a key feature in LaMonica’s description of empathy: “Empathy involves an accurate perception of the client’s world by the helper, communicating this understanding to the client, and a healthcare provider who has an empathic understanding of the patient but does not communicate such an understanding would not be perceived as an empathic healthcare provider” (Ward et al., 2012; Motataianu, 2014). Intention to help is another specific feature of empathetic engagement in patient care (Kourakos et al., 2018). Understanding in itself does not necessarily imply that the individual is compelled to help. However, readiness to respond to another person’s call for help is indeed synonymous to help (Rohani et al., 2018).

Despite the compelling evidence highlighting the importance of empathy in patient care, to date, evidence shows a trending erosion in empathy in medical practice and in healthcare provider and patient relationships coupled with the current lack of existing effective mechanisms with which to promote healthcare providers’ empathy. Recognizing a decline in empathy indicates a call to action to identify effective methods to improve healthcare providers’ expressed empathy. An integrated approach to empathy training involving storytelling through self-reflection, empathy matching card games, empathy virtual patient and toy video, role-play, and simulation are rarely utilized in health profession training interventions, despite evidence recommendations to implement them (Ward et al., 2012; Williams et al., 2015a; Bauchat et al., 2016). The intervention protocol for this study was developed based on the standardized empathy-focused training of Brett Williams and Jessica Delano’s empathy interventions (Williams, 2014; Williams et al., 2014a; Holden, 2017). This intervention was conducted for three consecutive days by one psychologist and one psychiatrist using brief PowerPoint presentations on the meaning of empathy, empathy matching cards, empathy-video show, storytelling, and role-playing, video show on a virtual patient, empathy toy, and the critical steps of ways how to improve empathy.

Educators in the health professions have uncritically adopted the concept of empathy, and it fits poorly with the clinical reality in healthcare provider–patient encounters (Rohani et al., 2018). Recent evidence confirmed that the culture of medicine and medical education might be such that empathy is undervalued and under-taught (Rohani et al., 2018). A wealth of research addresses intraindividual determinants of pain, distress, suffering, and disability. In contrast, limited attention has been devoted to the interpersonal domain. When confronted with the suffering of others, a variety of responses are elicited, ranging from ignoring to distress, empathy, and inclinations to comfort or assist. We cannot understand the disease without understanding the patient. The clinician cannot fully understand the patient without entering into the patient’s world on the bridge of empathy (Goubert et al., 2005).

Nowadays, there is a dramatical attenuation of empathy throughout clinical training, course of healthcare, and education leading to poor communication patterns in practicing healthcare providers partly due to a lack of training in empathy (Bauchat et al., 2016). Moreover, the environment of healthcare and the educational process place an excessive emphasis on technological competence, placing more emphasis on treating the disease rather than on restoring the emotional wellbeing of the patients (Sinclair et al., 2017). The increased reliance on computer-based diagnostic and therapeutic technology by healthcare providers and the healthcare system may limit practitioners’ understanding of the significance of human connection and empathic engagement in patient care (Hojat, 2016d). Patients become dissatisfied with the health system when healthcare providers fail to provide them with emphatic care and a good relationship. This makes it less likely that patients will seek timely medical care, which has a direct impact on their wellbeing (Yang et al., 2014; Terezam et al., 2017).

As the literature indicated, there is no study conducted for assessing the effect of empathy-focused training interventions on the level of empathy of healthcare providers delivering care for women with obstetric fistulas in Ethiopia. Therefore, this study aimed to assess the effect of the empathy training intervention on the level of empathy of healthcare providers providing care for women with obstetric fistulas in Ethiopia. We hypothesized that empathy training has a significant positive change in the total mean empathy score of healthcare providers providing care for women with fistulas. This means that empathy training may significantly change the perspective-taking, compassionate care, and walking in patients’ shoes empathy domains of healthcare providers providing care for women with fistulas. Hence, the implementation of this empathy training intervention package for healthcare providers providing care for women with obstetric fistulas may improve their empathy for their future empathic clinical practice, enhanced interpersonal relationships, better patient outcomes, and satisfaction with care. Studying empathy and intervention to promote it is of paramount importance for improving the relationship between healthcare providers and fistula patients as this group of women have been devastated by their fistula condition and they need a more empathetic relationship with their care providers. Evidence also suggests that empathy is one of the most frequently reported humanistic components of patient care, a royal road to treatment, and an important component of professionalism in medicine and healthcare (Kourakos et al., 2018).

## Materials and methods

### Study area, design, and period

**Figure 1:**
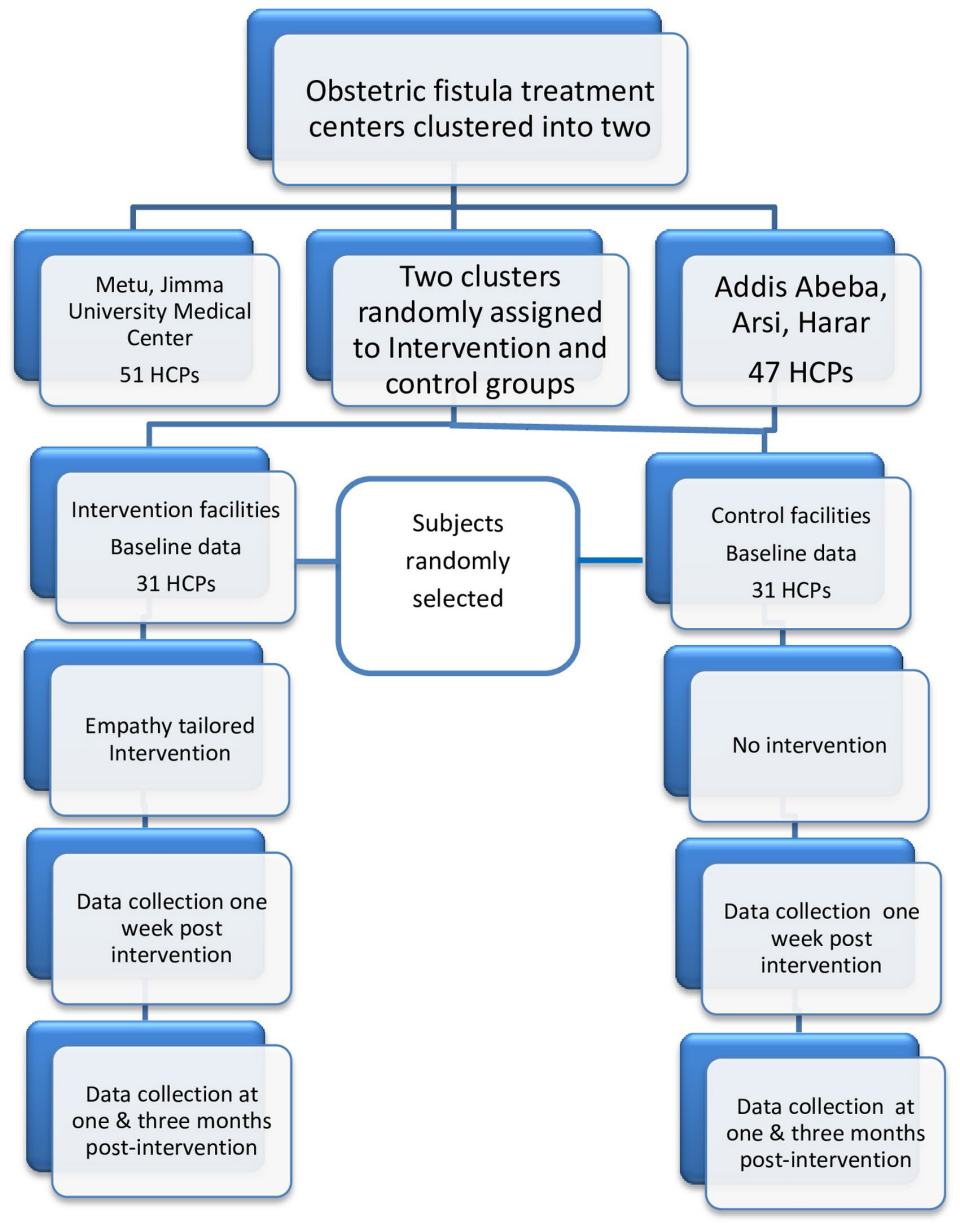
Schematic presentation of the sampling technique of the study.

A facility-based cluster randomized controlled trial pre–posttest interventional study was conducted from 20 December 2021 to 20 March 2022 at five obstetric fistula treatment centers in Ethiopia: Jimma University Medical Center, Asella Hospital, Harar, Mettu, and Addis Ababa Hamlin fistula centers. The Addis Ababa Fistula treatment center is the head office for other Hamlin fistula treatment centers and has a higher flow of fistula patients when compared with other treatment centers. The quotas of the healthcare providers recently providing care for women with an obstetric fistula at each facility are as follows: Jimma university medical center = 37, Mettu = 14, Addis Ababa Hamlin fistula = 32, Harar = 8, and Arsi = 7. Cluster randomization was chosen for practical reasons and to prevent the contamination introduced through participant selection (selection bias). Clusters were obstetric fistula treatment centers, which were matched by geographical area, the size of clinics, and the number of clinicians, and the paired units were randomly assigned to intervention or control groups. The population of women with fistulas at each treatment center have a similar background; most of them are from the countryside and with similar socio-economic features. To account for the differences in the size of clinics and the number of clinicians across each treatment center, initially, each treatment center was purposively allocated into two clusters based on their geographical area, size of clinics, and number of clinicians for maintaining the balance. The Jimma University Medical Center and Mettu were purposively selected as the first cluster, and the other remaining centers as the second cluster (see Figure 1). Then, two clusters were randomly assigned to the intervention and control arms, and the clinicians from each cluster were randomly selected.

### Source population

All clinicians (indicating all healthcare providers providing care for women with obstetric fistulas) delivering care for women with an obstetric fistula at five fistula treatment centers in Addis Ababa and Oromia Region were source populations.

### Study population

The study population comprised randomly selected healthcare providers available at five fistula treatment centers who provided care for women with obstetric fistula during data collection time and fulfilled the inclusion criteria.

### Inclusion criteria

All randomly selected healthcare providers who deliver care to women with an obstetric fistula at the center for at least a week before the actual study period (since this time is considered as the time of readiness for actual practice; Riess, 2018) were included in the study. The healthcare providers had to be aged between 19 and 65 years, and both sexes were included in the study.

### Exclusion criteria

Healthcare providers who were not able to participate consecutively in the training, who did not volunteer to participate, and who were not able to continue until the end of the study were excluded.

### Sample size determination

The recommended sample size for studies that planned to use the validated Jefferson Scale of Empathy (JSE) was 25 (Hojat et al., 2018b), but many studies showed significant improvements in empathy scores with varying sample sizes ranging from *n* = 10 to *n* = 263 (Brunero et al., 2010). Recent systematic reviews reported on the effect sizes attributed to an effective empathy intervention involving the standardized patient and simulated education using the Jefferson Scale of Empathy ranged from medium (0.5) to large effect (0.8), respectively (Shin et al., 2015; Cant and Cooper, 2017; Davison et al., 2017; Riess, 2018). Effect size (d), according to Cohen, is a measure of the magnitude of the effectiveness of a particular intervention (Cohen, 1988). Using Cohen’s values for effect sizes that have been widely used today (0.2 small, 0.5 medium, and 0.8 large) (Cohen, 1988) and the above current systematic review evidence for the effect of empathy interventions from similar previous empathy training interventions, the maximum sample size was calculated using the effect sizes 0.5 and 0.8 by using G* power 3.1.9.2 (with assumptions of the power of 80, *α* = 0.05, two tails, and an allocation ratio of 1) were 128 and 52, respectively. However, considering the intercluster correlation coefficient of 0.01 and considering the average cluster size and maximum cluster size for a restricted number of clusters, the design effect was calculated using the formula, Deff = 1 + ICC(ma-1), ma = average or maximum cluster size, using the average cluster size of (37 + 14 + 32 + 8 + 7/5) =19.6, Deff = 1.19, and then, Nc = Deff*N = 1.19*52 = 62 (31 in intervention arm and 31 in control arm).

### Sampling technique

**Figure 2:**
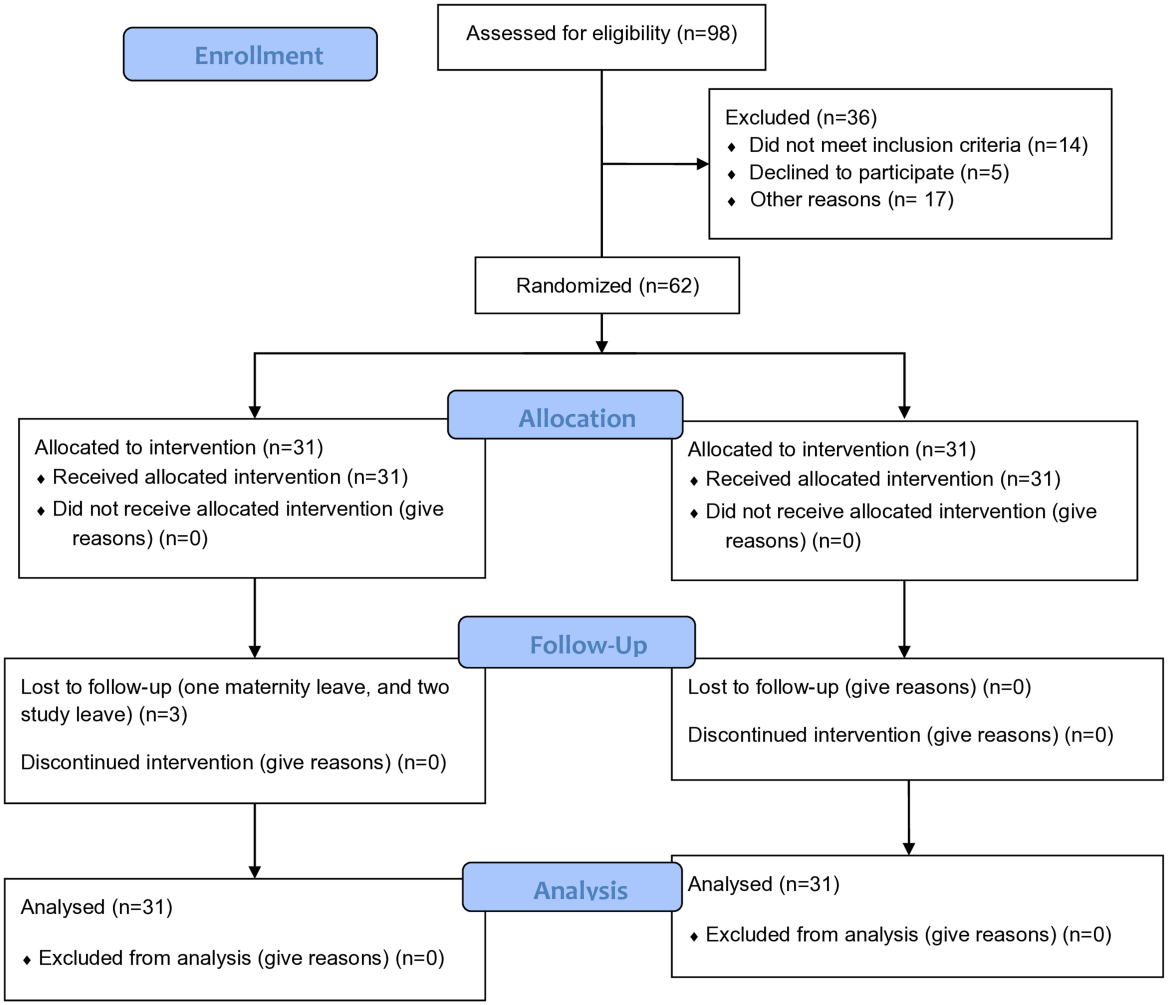
Participant flow diagram (CONSORT 2010 flow diagram).

Initially, the five obstetric fistula treatment sites were clustered into two purposively based on their geographical areas, their size of clinics, and the number of clinicians to maintain a balance between each cluster and to avoid the contamination of information; then the two clusters were randomly assigned to the intervention and control groups using lottery methods. From each cluster, consecutive serial numbers (codes) were assigned for all participants, and using that code, the predetermined numbers of study participants were randomly selected using a computer-generated random number by an investigator while considering the preset eligibility criteria (Figures 1,2).

### Outcome

Mean score changes in the level of empathy of healthcare providers measured before the training, and one week, one month, and three months after the training were the outcome measures in this trial.

### Data collection tools and procedures

The data collection tool was adopted from the Jefferson Scale of Empathy–Health Professions version (JSE-HP-S) developed with 20 Likert-type items answered on a 7-point scale (1 = strongly disagree to 7 = strongly agree), which allows for more variation and thus more precise discriminatory power. To decrease the confounding effect of the “acquiescence response style” (e.g., the tendency to constantly agree or disagree with yea-sayers and nay-sayers), a balance was maintained by making 10 items positively worded and 10 items negatively worded. Half of the items are directly scored according to their Likert weights (1 = strongly disagree, 7 = strongly agree) and the other half are reversely scored (1 = strongly agree, 7 = strongly disagree) (Hojat et al., 2018b).

Based on the underlying confirmed factor structure of the JSE, there are three component structures for the 20 JSE items. Component one is perspective-taking, a component of the JSE that has been described as the core cognitive ingredient of empathy, which contains 10 items including understanding makes patients feel better, understanding body language in communication, sense of humor and clinical outcomes, standing in patients’ shoes, understanding is therapeutic to a patient, non-verbal cues and body language in understanding patients, empathy and clinical success, understanding emotions in patient–healthcare provider relationship, thinking like patients for better care, and empathy. Component two is compassionate care with items understanding patients’ feelings influencing treatment, attention to patients’ emotions, attention to patients’ personal experience, patient–healthcare provider emotional ties in medical treatment, life events in understanding physical complaints, place of emotion in medical treatment, healthcare providers influenced by patients’ bonds, and enjoying literature and arts; and component three is walking in patients’ shoes with items viewing patients’ perspectives and taking patients’ perspectives (Hojat, 2016b,c; Terezam et al., 2017; Hojat et al., 2018b).

Studies provide strong evidence in support of the psychometric soundness of the JSE in different samples of health professions, in a variety of health profession disciplines, and in different countries with different educational systems and cultural values. Consistencies in most of the major findings in the studies generally show that the reliability coefficients of the JSE are in the 0.70s and 0.80s (M = 0.78), a well-acceptable range for psychological tests (Alcorta-Garza et al., 2016).

Before data collection, the protocol for the study was reviewed and approved by the Institutional Review Board of Jimma University (Ref. No: IRB 000281/2019). All participants signed an informed consent form before participation. All study procedures followed the relevant guidelines and regulations of the Declaration of Helsinki. Five MSc Midwifery students and one senior MSc Midwife as a supervisor collected data through a self-administered questionnaire.

### Data analysis

The collected data were entered into Epi data version 3.5.3 (Odense Denmark) for double data entry verification. Then, the data were coded, cleaned, and exported to the International Business Machines Corporation Statistical Product and Service Solutions (IBM SPSS) version 23 for further cleaning and analysis. Descriptive statistics such as frequency, mean, standard deviation, and percentage difference were analyzed in the aggregate for each arm. To account for the repeated measures, clustering effects, effects of potential covariates on change in empathy scores, and baseline participants variability; and to measure the independent effect of the empathy intervention, a linear mixed effects model was fitted. Initially, assumptions such as homoscedasticity and normality assumptions were tested and satisfied. Data from the widest form were restructured to its longest form based on the IDs of participants and empathy score as trans for each follow-up time empathy score (prescore, after a week score, after a month score, and after three months score). The linear mixed effects models were fitted for testing the fixed effects of the socio-demographic characteristics of participants on the mean empathy scores between control and intervention arms. For avoiding model convergence, we considered only the main effects. Empathy score was used as the dependent variable; age, sex, marital status, religion, profession educational level, work experience, and training on compassionate and respectful care as fixed effects; time as a random effect; and baseline empathy score as covariates in the primary model. After conducting a fixed effect model for participants’ baseline characteristics, variables such as age, profession, work experience, and training on compassionate and respectful care were found statistically significant for the baseline empathy score of the control arm, while there were no significant socio-demographic factors for the baseline empathy score of the intervention arm. To fit the final model, we excluded all variables that did not show a significant effect in the primary model (sex, marital status, religion, and educational level). Then, after controlling for these variables in the model, a final linear mixed effects model was fitted for the intervention arm considering the restricted maximum likelihood estimation, and including both the intercept and random slopes. Akaike’s information criterion (AIC) with a small value was considered when the model was fitted. An independent *t*-test was used for comparing the mean scores of the intervention group with the control group. Paired *t*-tests and average percentage differences were used for subgroup data analysis. Intention-to-treat analysis was conducted based on the participants’ baseline and subsequent follow-up data. Cohen’s d-effect size was analyzed after standardizing the mean scores to Z-scores. Statistical significance was declared at *p* < 0.05.

### Intervention description

This intervention protocol/empathy training is an intervention package that was developed based on the standardized empathy-focused training of Brett Williams and Jessica Delano empathy interventions (Williams, 2014; Williams et al., 2014a; Holden, 2017). Professor Brett Williams designed the intervention package to run for 90 minutes, including 10 minutes for completion of the “before” survey [Jefferson Scale of Empathy–Health Professions version (JSE-HP-S)] while suggesting that timeframes can be adjusted by the researcher if he/she wishes to use additional contents in the package in sessions longer than 90 minutes (Williams, 2014; Williams et al., 2014a). Evidence suggests that the skills required for emphatic patient care or empathy as a learned behavior can be taught and maintained with training programs lasting as short as one hour (Guastello and Frampton, 2014; Williams et al., 2015b). However, this intervention was conducted for three consecutive days. The training was only for the intervention arms. One psychologist and one psychiatrist carried out the intervention with PI as a facilitator. There were eight sessions in this intervention. The intensity of the intervention was five hours per day. The detailed sequence of steps followed for the intervention were: 1. Pre-test survey (before intervention) (10 minutes). 2. Brief PowerPoint presentation and video show concerning the meaning of empathy in the context of patient care (one hour). 3. Empathy matching cards to consider the nuances of the different terms and consider what this might mean for different healthcare professions and to ensure the group has the same understanding when they use the term ‘empathy’ (one hour). 4. Storytelling and role-playing by some participants on their encounters with women with obstetric fistulas (one hour). 5. Video show on virtual patient and empathy toy video (one hour). 6. “If I was the patient activity,” participants were asked if they were the patient in the video simulation, and how they would feel and to identify missteps and compromised values in the video (20 minutes). 6.1. Asking questions for discussion on how those video simulations/narratives relate to their own lives in providing care for women with obstetric fistulas (10 minutes). 7. Wrap-up video show regarding the critical steps of ways how to improve empathy (30 minutes). 8. Post-intervention survey at one week, after a month, and three months (10 minutes) (see Supplementary Tables S1, S2).

## Results

Among the 62 randomly selected participants, 31 were controls and 31 were in the intervention arm with a 100% response rate. The mean age of the control and intervention groups was 38.81 ± 12.76 and 34.23 ± 3.92 years, respectively. More than three-fourths of the controls and close to two-thirds of the intervention groups were female participants. In both groups, most of the participants were first-degree holders in educational status, nurses in the profession, and married in marital status (Table 1).

**Table 1:** Socio-demographic characteristics of study participants at five fistula centers, in Ethiopia, from 20 December 2021 to March 2022 (*n* = 62).

Variables	Descriptor	Control (*n* = 31)	Intervention (*n* = 31)	Baseline empathy score mean (SD)			
		*n* (%)	*n* (%)	Control	*p*-value	Intervention	*p*-value
Age (mean, SD)		38.81 ± 12.76	34.23 ± 3.92	29.23	0.001		0.479
Sex	1. Male	7 (22.6)	12 (38.7)	93.42 (5.62)	0.083	100.78 (10.70)	0.518
	2. Female	24 (77.4)	19 (61.3)	106.53 (8.08)		93.42 (12.32)	
Profession	1. Midwifery	4 (12.9)	11 (35.5)	79.25 (11.22)	0.005	104.16 (10.93)	0.724
	2. Nurse	15 (48.4)	18 (64.5)	117.14 (6.07)		98.99 (11.95)	
	3. HO	1 (3.2)		81.42 (19.67)			
	4. Doctor	4 (12.9)	2 (6.5)	92.39 (9.45)		88.13 (19.27)	
	5. Other^a^	7 (22.6)		129.68 (9.34)			
Marital status	1. Never married	7 (22.6)	5 (16.1)	107.30 (7.07)	0.850	103.72 (13.60)	0.900
	2. Married	21 (67.7)	23 (74.2)	99.26 (6.61)		103.85 (7.90)	
	3. Cohabiting		1 (3.2)	94.00 (18.20)		87.82 (28.88)	
	4. Divorced	3 (9.7)	2 (6.5)	99.35 (10.43)		92.99 (17.57)	
Religion	1. Orthodox	20 (64.5)	14 (45.2)	89.82 (7.21)	0.120	96.50 (10.13)	0.890
	2. Protestant	9 (29)	14 (45.2)	94.09 (7.61)		93.78 (11.10)	
	3. Muslim	2 (6.5)	3 (9.7)	116.02 (9.87)		101.01 (16.94)	
Educational status	1. Diploma	7 (22.6)	4 (12.9)	101.63 (8.49)	0.083	100.07 (14.08)	0.915
	2. 1^st^ degree (BSc)	8 (25.8)	19 (61.3)	105.91 (8.99)		93.80 (12.43)	
	3. Medical doctor/1^st^ degree	8 (25.8)		83.03 (8.06)			
	4. 2^nd^ degree (MSc)	3 (9.7)	6 (19.4)	97.97 (11.12)		90.98 (15.90)	
	5.Gyne/obstetrics 2^nd^ degree	3 (9.7)	2 (6.5)	78.00 (9.05)		103.54 (20.11)	
	6. Ph.D. and above	2 (6.5)		133.33 (19.75)			
7. Years of work experience (mean, SD)		6.71 ± 3.78	10.23 ± 15.70	10.23/10.10	0.016		0.449
8. Attended CRC training	1. Yes	7 (22.6)	10 (32.3)	90.22 (7.89)	0.020	97.58 (11.38)	0.918
	2. No	24 (77.4)	21 (67.7)	109.73 (6.11)		96.61 (10.81)	

a Lab technologist, physiotherapist, and psychiatrist.

### Level of empathy scores of study participants

**Figure 3:**
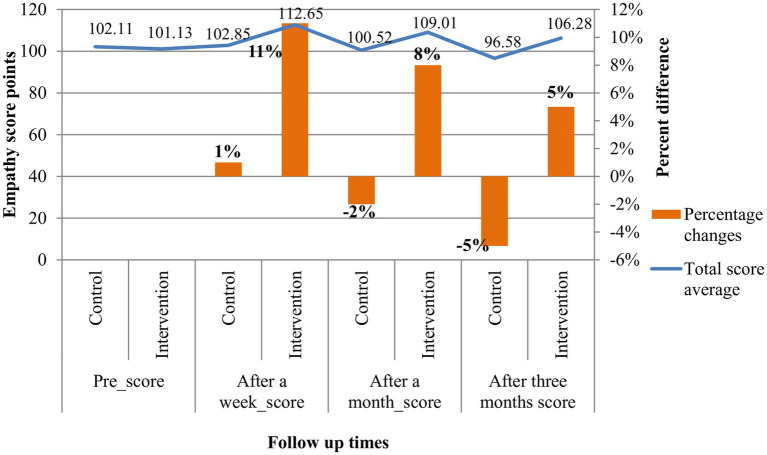
Empathy scores pre- and post-intervention among healthcare providers at five fistula centers, in Ethiopia, from 20 December 2021 to March 2022.

The total average baseline empathy scores of both groups were similar. There was a significant difference in the baseline empathy score of the control arm across their age, profession, work experience, and training on compassionate and respectful care. In contrast, the baseline empathy score of the intervention arm had no statistically significant difference for the participants’ socio-demographic features (Table 1). After a week, a month, and three months of post-intervention, the total mean scores of the intervention group were 112.65, 109.01, and 106.28 with overall percentage changes of 11, 8, and 5% from the baseline scores, respectively. The total mean scores of the control group were in a decreasing trend, that is, 102.85, 100.52, and 96 after a week, a month, and three months post-intervention with overall percentage changes of 1%, −2%, and − 5% from prescores, respectively. Post-intervention, changes in the empathy level of healthcare providers in terms of total mean score differences, and percentage changes across each follow-up period were higher among the intervention arms (Figure 3).

After a week post-intervention, 10 items: item one (healthcare providers’ understanding of women with fistulas’ feelings and the feelings of their families does not influence obstetric fistula treatment outcomes), item three (it is difficult for a healthcare provider to view things from women with fistulas’ perspectives), item six (because people are different, it is difficult to see things from women with fistulas’ perspectives), item seven (attention to women with fistulas’ emotions is not important in their interview), item eight (attentiveness to women with fistulas’ personal experiences does not influence obstetric fistula treatment outcomes), item 11 (women with fistulas’ illnesses can be cured only by targeted treatment; therefore, healthcare providers’ emotional ties with them do not have a significant influence in obstetric fistula treatment outcomes), item 12 (asking women with fistula about what is happening in their lives is not helpful in understanding their physical complaints), item 14 (I believe that emotion has no place in the treatment of obstetric fistula), item 18 (healthcare providers should not allow themselves to be influenced by strong personal bonds between women with fistula and their family members), and item 19 (I do not enjoy reading non-medical literature or the arts) showed higher percentage changes greater than 20%. After a month of intervention, seven items: items one, seven, eight, 12, 14, 15 (empathy is a therapeutic skill without which a clinician’s success is limited), and 19 showed higher percentage changes greater than 10%, but after three months of follow-up, only four items: items one, 12, 13 (healthcare providers should try to understand what is going on in women with fistulas’ minds by paying attention to their non-verbal cues and body language), and 15 showed a percentage change higher than or equal to 10% (Table 2).

**Table 2:** Empathy average scores pre- and post-intervention displayed percentage differences among study participants at five fistula centers, in Ethiopia, from 20 December 2021 to March 2022 (*n* = 62).

Empathy domains	Items	Pre-score		After a week score		Percentage changes		After a month score		Percentage changes		After a 3 months score		Percentage changes	
		Contr	Interv	Contr	Interv	Contr	Interv	Contr	Interv	Contr	Interv	Contr	Interv	Contr	Interv
Perspective taking	2	6.65	6.45	6.45	6.03	−3%	−7%	6.52	6.65	−2%	3%	6.19	6.84	−7%	6%
	4	6.68	6.16	6.16	5.42	−8%	−12%	5.10	6.68	−24%	8%	6.00	6.71	−10%	9%
	5	6.29	6.00	6.00	5.19	−5%	−14%	5.97	6.29	−5%	5%	6.00	6.26	−5%	4%
	9	6.65	6.06	6.06	6.06	−9%	0%	6.03	6.65	−9%	10%	5.87	6.55	−12%	8%
	10	6.68	6.29	6.29	5.77	−6%	−8%	5.65	6.68	−15%	6%	6.10	6.65	−9%	6%
	13	6.35	6.03	6.03	5.84	−5%	−3%	5.84	6.35	−8%	5%	6.03	6.61	−5%	**10%**
	15	6.52	5.61	5.61	5.00	−14%	−11%	5.00	6.52	−23%	**16%**	5.61	6.16	−14%	**10%**
	16	6.48	6.55	6.55	5.90	1%	−10%	5.90	6.48	−9%	−1%	6.55	6.65	1%	2%
	17	6.42	6.16	6.16	5.65	−4%	−8%	5.65	6.42	−12%	4%	6.16	6.48	−4%	5%
	20	6.84	6.55	6.55	6.77	−4%	3%	6.77	6.84	−1%	4%	3.77	6.90	−45%	5%
Compassionate care	1	3.81	3.39	4.61	6.03	21%	**78%**	3.71	4.19	−3%	**24%**	3.39	4.81	−11%	**42%**
	7	3.19	4.10	3.90	6.03	22%	**47%**	3.81	4.81	19%	**17%**	3.71	4.26	16%	4%
	8	3.32	4.13	3.87	5.06	17%	**23%**	4.32	4.68	30%	**13%**	3.94	4.06	19%	−2%
	11	3.94	4.29	3.71	5.61	−6%	**31%**	4.77	4.06	21%	−5%	4.29	4.00	9%	−7%
	12	2.87	4.52	3.48	6.10	21%	**35%**	4.45	5.13	55%	**14%**	4.13	5.03	44%	**11%**
	14	2.97	4.03	4.16	6.03	40%	**50%**	4.61	5.03	55%	**25%**	4.03	4.32	36%	7%
	18	4.87	3.65	4.35	4.74	−11%	**30%**	5.03	3.13	3%	−14%	3.65	3.06	−25%	−16%
	19	3.16	4.23	3.84	5.65	22%	**34%**	4.90	4.84	55%	**14%**	4.23	4.06	34%	−4%
Walking in patients’ shoes	3	3.87	3.77	4.23	5.29	9%	**40%**	2.97	4.13	−23%	10%	3.77	3.68	−3%	−2%
	6	4.55	3.16	4.84	4.48	6%	**42%**	3.52	3.45	−23%	9%	3.16	3.19	−31%	1%
	Total score	102.11	101.13	102.85	112.65	1%	11%	100.52	109.01	−2%	8%	96.58	106.28	−5%	5%

Contr-control group, Interv-intervention group. Bold value: emphasize percentage changes of some items across follow-up times.

Throughout each subsequent follow-up period, the mean empathy score differences of the intervention arm showed statistically significant changes with declining order (Table 3).

**Table 3:** Empathy score changes at different intervals in the post-intervention period among study participants at five fistula centers, in Ethiopia, from 20 December 2021 to March 2022 (*n* = 62).

Follow-up periods (intervals)	Groups	Paired differences		*t*	*p*-value	95% CI of the difference	
		Mean	Standard deviation			Lower	Upper
After a week	Control	0.77	13.34	0.32	0.749	−4.12	5.67
	Intervention	11.55	26.39	2.44	0.021	1.87	21.23
After a month	Control	−1.58	18.34	−0.48	0.635	−8.31	5.14
	Intervention	7.87	14.17	3.09	0.004	2.67	13.07
After three months	Control	−5.52	26.27	−1.17	0.252	−15.15	4.12
	Intervention	5.16	10.53	2.73	0.011	1.30	9.02

The effect of empathy training on the total mean empathy score changes of the intervention arm compared to the control arm at each follow-up time showed a statistically significant difference with increasing effect size. After a week of intervention: the intervention arm mean score was 112.65 ± 18.99, the control arm mean score was 102.85 ± 15.65, d = 0.55, *p* = 0.03; after a month of intervention: the intervention arm mean score was 109.01 ± 17.79, the control arm mean score was 100.52 ± 12.57, d = 0.53, and *p* = 0.034; and after three months of intervention: the intervention arm mean score was 106.28 ± 16.24, the control arm mean score was 96.58 ± 14.69, d = 0.60, and *p* = 0.016 (Table 4).

**Table 4:** Total mean empathy score changes and effect sizes pre- and post-intervention among study participants at five fistula centers, in Ethiopia, from 20 December 2021 to March 2022 (*n* = 62).

Groups		Mean (± SD)	Percentage changes	t	P-value	Effect size(d)	95% CI Difference	
							Lower	Upper
Pre-score	Intervention	101.13 (17.67)		−0.23	0.819	−0.06	−0.57	0.45
	Control	102.10 (15.38)						
After a week score	Intervention	112.65 (18.99)	11%	2.22	0.030	0.55	0.05	1.04
	Control	102.85 (15.65)	1%					
After a month score	Intervention	109.01 (17.79)	8%	2.17	0.034	0.53	0.04	1.03
	Control	100.52 (12.57)	−2%					
After 3 months score	Intervention	106.28 (16.24)	5%	2.47	0.016	0.60	0.11	1.09
	Control	96.58 (14.69)	−5%					

## Discussion

In this trial of empathy training, the effect of the intervention on the empathy level of the intervention arm compared to the control arm had a statistically significant difference in their empathy mean scores with a medium effect size. The total mean empathy scores of the intervention arm were 112.65, 109.01, and 106.28 with overall percentage changes of 11%, 8%, and 5% after a week, a month, and three months of intervention from the baseline scores, respectively. These changes in the mean scores of healthcare providers’ empathy levels attributed to the effect of empathy training were tested and found to be significantly different from those of the control arm.

In this study, the pre-intervention empathy level of healthcare providers was lower than the national and international study report with JSE scores (Kommalage, 2011; Hegazi and Wilson, 2013; Hojat et al., 2013; Park et al., 2015; Williams et al., 2015a; Hojat et al., 2018a; Kataoka et al., 2019), but consistent with one similar study both in design and measurement with JSE (Ahmadzadeh et al., 2019). This indicates the declining level of empathy among healthcare providers in this study, which might be because of healthcare providers being overburdened in clinical practice and a lack of adequate time for empathetic concerns of patients and perspective-taking. This is supported by previous studies showing that a decline in empathy is associated with an adaptive response to increased responsibility and workload among practicing healthcare providers, which resulted in lower medical regard and empathy for specific stigmatized patient groups (Boyle et al., 2010; Williams et al., 2012a,b, 2013, 2014b, 2015a). This also collaborates with a recent study which indicates that, a long working hours for healthcare providers results in negative patient-related outcomes and adverse clinician–patient interactions and interpersonal interactions among colleagues (Grover et al., 2018).

In this trial, the empathy level of healthcare providers providing care to women with obstetric fistula was increased from the prescore of 101.13 to 112.65 total mean score after one week of intervention. This showed that the empathy score of healthcare providers increased by 11% after a week of the empathy training intervention. Of the 20 items on the scale, half of the items’ scores increased pre- to post-survey by having a greater than 20% difference in scores. This showed that at one week of intervention, the empathy level of healthcare providers was increasing almost within the eight items of the compassionate care component of empathy than other empathy component items but with declining trends after a month and three months post-intervention. This is because the emotional components of empathy are increasing soon after training but are less sustainable over time than the cognitive part of empathy. Previous studies confirmed that when affective empathy is automatically elicited (Lamm et al., 2007), the affective element prevails over cognitive function, which gives a fuller and more meaningful relationship experience between the empathizer and the target (Kerem et al., 2001) and, at the same time, imparts energy for the empathizer to help the target (Håkansson and Montgomery, 2003). Evidence avers this that perspective-taking or placing oneself in the position of others is not easy. It requires first compassion for other people to be able to walk in patients’ shoes and perform at the best level, despite internal and external difficulties (Undung and De Guzman, 2009). Similarly, items one and 14 of the compassionate care domain of empathy had the greatest difference with a 78% and 50% increase in score, respectively. These two items remained to have greater than 20% differences in scores after a month of intervention. These items, in particular, are consistent with the essence of empathy in the intent to understand another’s emotions, feelings, concerns, and perspectives (Hojat, 2016a,b; Hojat et al., 2018b). Unfortunately, negative changes in the percentage differences on items two, four, five, 13, 15, and 17 demonstrated a need to increase healthcare providers’ understanding of the significance of body language and non-verbal cues, the therapeutic essence of empathy, the importance of having a sense of humor, and thinking like patients on the patient experience during clinical encounters.

After three months of intervention, the total mean score in the empathy of healthcare providers in the intervention arm was 106.28. This indicated a 5% increase in the empathy score of healthcare providers after three months of empathy training intervention. At this time, from the 20 items on the Jefferson Scale of Empathy, 14 items had a positive percentage change of greater than 4%. Similarly, item one remained to have the greatest percentage difference of 42%. Items three, eight, 11, 18, and 19 had negative percentage changes. This indicates a need to sustain healthcare providers’ attention to patients’ personal experiences, patient–healthcare providers’ emotional ties, and the need of reading non-medical literature or the arts, respectively. Furthermore, a probable reason for a 5% dropped empathy score among the control arm may be due to the differences in terms of the patients they see or patient flows, ages, work experiences, and exposure to compassionate care training from the intervention arms.

In this trial, the post-intervention empathy score of healthcare providers increased after a week of follow-up but with decreasing trends after a month and three months post-intervention. This may be, as one infers, from the percentage changes seen on the Jefferson Scale of Empathy items, items with emotional components of empathy are more responsive to the intervention initially and less sustainable than items with the cognitive components of empathy, suggesting the need for sustained empathy training and integration with educational and training curriculums to promote the empathy level of healthcare providers. This is consistent with a previous randomized prospective study on the outcomes of an empathy intervention, which shows an increase in empathy levels immediately within seven days post-intervention following a three-day simulation experience but a decreased empathy level 90 days post-intervention (Lor et al., 2015).

The effect size of empathy training in the intervention arm was increased from one week of post-intervention 0.55 to 0.60 after three months of intervention. This indicates a medium change in effect size. This is higher than previous studies’ reports on changes in the effect size of empathy levels from the empathy intervention from 0.18 to 0.30 (Williams et al., 2015b; Holden, 2017; Riess, 2018) but consistent with recent systematic review reports on effect sizes attributed to mean scores from empathy interventions involving the standardized patient and simulated education using the Jefferson Scale of Empathy ranged from the medium (0.5) to large effect (0.8), respectively (Shin et al., 2015; Cant and Cooper, 2017; Davison et al., 2017; Riess, 2018). This is because our recent empathy training intervention package incorporates some of a similar training package with additional training resources.

### Strengths

This trial was completed successfully from both a methodological and practical point of view and is generalizable to all healthcare providers providing care for women with obstetric fistulas in Ethiopia.

To the best knowledge of the investigators, this is the first trial study to assess the effect of empathy training on the levels of empathy of healthcare providers providing care for women with obstetric fistula. This finding provides a starting point for researchers to conduct further research on the topic; for healthcare providers to demonstrate empathy in clinical practice; and for policymakers to emphasize the importance of empathy and design strategies on how to sustain and include empathy in training programs.

The study is a cluster randomized controlled trial, which reduced the contamination of the information and assessed the effect of the intervention. The intervention used different training packages. The study assessed the trends in the effect of the intervention at different follow-up times. Intention-to-treat analysis was used to compensate for losses to follow-up data. The most reliable JSE, which has enjoyed broad international attention, was used for data collection.

### Limitations

A relatively small sample size was used due to feasibility issues which made us unable to use advanced statistical analyses. The demand characteristics and the nature of measurement, which were self-reported on the JSE, were the other limitations of this study. There may be selection bias at the point of participant selection due to the inability to include all participants identified as eligible. Finally, our focus was to look at the level of empathy of healthcare providers providing care to women living with obstetric fistulas and to see the effect of empathy training on their empathy scores. Further research is required to investigate the effect of such training on other healthcare providers with larger populations and longer follow-up times. Future studies should also assess the effect of empathy training on healthcare providers’ professional job satisfaction, patient treatment outcomes, and adherence to healthcare services.

## Conclusion

In this trial, the empathy training intervention was effective by having statistically significant difference changes in the post-intervention total mean empathy scores of healthcare providers. The intervention was found to have more than a medium effect size. Even though the effect of the intervention on the empathy of healthcare providers was found effective with a medium effect size, the total means empathy scores of the healthcare providers showed a decreasing trend after a month and three months follow-up time. This indicates the need for sustained empathy training and integration with educational and training curriculums to promote the empathy level of healthcare providers. Researchers should increase the duration and dose of the recent empathy training package for sustained effects. Practitioners, tutors, and program designers should incorporate empathy training packages into their teaching and training curriculums. Healthcare providers providing care for women with obstetric fistula need to have emotional ties with their patients; pay attention to patients’ personal experiences; understand the significance of body language and non-verbal cues, the therapeutic essence of empathy, the importance of having a sense of humor, and thinking like the patients on the patient experience during clinical encounters; and practice empathetic engagement in all circumstances of patient care. Moreover, understanding clinicians’ empathy levels and effective ways to promote their empathy is significantly important and can direct practice, program and strategy change, and future studies. There should be a multicenter cluster randomized control trial to evaluate the effect of empathy training on the empathy level of all practicing healthcare providers in Ethiopia.

## Data availability statement

The original contributions presented in the study are included in the article/Supplementary material, further inquiries can be directed to the corresponding author.

## Ethics statement

The studies involving human participants were reviewed and approved by Institutional Review Board of Jimma University (Ref. No: IRB 000281/2019). The patients/participants provided their written informed consent to participate in this study. This study followed the updated CONSORT extension checklist for reporting a cluster randomized trial (Campbell et al., 2012) (see Supplementary Table 3).

## Author contributions

BFH and ZBK conceived the concept. BFH designed, implemented, supervised the fieldwork, analyzed the data, and wrote the manuscript. ZBK and LSD were involved in the design of the study and data analysis and critically revised the manuscript. All authors critically read and approved the submission of the final version of the manuscript for publication.

## Conflict of interest

The authors declare that the study was conducted in the absence of any commercial or financial relationships that could be construed as a potential conflict of interest.

## Publisher’s note

All claims expressed in this article are solely those of the authors and do not necessarily represent those of their affiliated organizations, or those of the publisher, the editors and the reviewers. Any product that may be evaluated in this article, or claim that may be made by its manufacturer, is not guaranteed or endorsed by the publisher.

## References

[ref1] Ahmadzadeh A. Esfahani M. N. Ahmadzad-Asl M. Shalbafan M. Shariat S. V. (2019). Does watching a movie improve empathy? A cluster randomized controlled trial. Canad. Med. Educ. J. 10:e4. doi: 10.36834/cmej.56979, PMID: 31807222 PMC6892313

[ref2] Alcorta-Garza A. San-Martín M. Delgado-Bolton R. Soler-González J. Roig H. Vivanco L. (2016). Cross-validation of the Spanish HP-version of the Jefferson scale of empathy confirmed with some cross-cultural differences. Front. Psychol. 7:1002. doi: 10.3389/fpsyg.2016.01002, PMID: 27462282 PMC4940391

[ref3] Bauchat J. R. Seropian M. Jeffries P. R. (2016). Communication and empathy in the patient-centered care model—why simulation-based training is not optional. Clin. Simul. Nurs. 12, 356–359. doi: 10.1016/j.ecns.2016.04.003

[ref4] Blair R. J. R. (2005). Responding to the emotions of others: dissociating forms of empathy through the study of typical and psychiatric populations. Conscious. Cogn. 14, 698–718. doi: 10.1016/j.concog.2005.06.004, PMID: 16157488

[ref5] Boyle M. J. Williams B. A. Brown G. T. Molloy A. M. McKenna L. G. Molloy E.. (2010). Levels of empathy in undergraduate health science students. Internet J. Med. Educ. 1, 1–14. doi: 10.5580/1b15#sthash.EzsawuHr.dpuf

[ref6] Brunero S. Lamont S. Coates M. (2010). A review of empathy education in nursing. Nurs. Inq. 17, 65–74. doi: 10.1111/j.1440-1800.2009.00482.x, PMID: 20137032

[ref7] Campbell M. K. Piaggio G. Elbourne D. R. Altman D. G. (2012). Consort 2010 statement: extension to cluster randomised trials. BMJ 345:e5661. doi: 10.1136/bmj.e5661, PMID: 22951546

[ref8] Cant R. P. Cooper S. J. (2017). Use of simulation-based learning in undergraduate nurse education: an umbrella systematic review. Nurse Educ. Today 49, 63–71. doi: 10.1016/j.nedt.2016.11.015, PMID: 27902949

[ref9] Clark A. (2010). Empathy and sympathy: therapeutic distinctions in counseling. J. Ment. Health Couns. 32, 95–101. doi: 10.17744/mehc.32.2.228n116thw397504

[ref10] Cohen J. (1988). Statistical power analysis for the behavioral sciences Ind cd. Hillsdale. NJ: Erihaum.

[ref11] Davison J. Mackay B. McGivern M. J. (2017). The potential of simulation to enhance nursing students’ preparation for suicide risk assessment: a review. Open J. Nurs. 7, 129–144. doi: 10.4236/ojn.2017.72012

[ref12] Goubert L. Craig K. D. Vervoort T. Morley S. Sullivan M. de CAC W.. (2005). Facing others in pain: the effects of empathy. Pain 118, 285–288. doi: 10.1016/j.pain.2005.10.025, PMID: 16289804

[ref13] Grover S. Sahoo S. Bhalla A. Avasthi A. (2018). Psychological problems and burnout among medical professionals of a tertiary care hospital of North India: a cross-sectional study. Indian J. Psychiatry 60:175. doi: 10.4103/psychiatry.IndianJPsychiatry_254_17, PMID: 30166673 PMC6102958

[ref14] Guastello S. Frampton S. B. (2014). Patient-centered care retreats as a method for enhancing and sustaining compassion in action in healthcare settings. J. Compassionate Health Care 1:2. doi: 10.1186/s40639-014-0002-z

[ref15] Håkansson J. Montgomery H. (2003). Empathy as an interpersonal phenomenon. J. Soc. Pers. Relat. 20, 267–284. doi: 10.1177/0265407503020003001, PMID: 37088974

[ref16] Hegazi I. Wilson I. (2013). Maintaining empathy in medical school: it is possible. Med. Teach. 35, 1002–1008. doi: 10.3109/0142159X.2013.802296, PMID: 23782049

[ref17] Hojat M. (2016a). “A definition and key features of empathy in patient care” in Empathy in health professions education and patient care. (Heidelberg, New York, Dordrecht, London: Springer, Cham), 71–81.

[ref18] Hojat M. (2016b). “Empathy and patient outcomes” in Empathy in health professions education and patient care. (Heidelberg, New York, Dordrecht, London: Springer, Cham), 189–201.

[ref19] Hojat M. (2016c). “Empathy as related to personal qualities, career choice, acquisition of knowledge, and clinical competence” in Empathy in health professions education and patient care. (Heidelberg, New York, Dordrecht, London: Springer, Cham), 151–167.

[ref20] Hojat M. (2016d). “Erosion and enhancement of empathy” in Empathy in health professions education and patient care. (Heidelberg, New York, Dordrecht, London: Springer, Cham), 203–234.

[ref21] Hojat M. (2016e). “Parting thoughts: a systemic paradigm of empathy in patient care and future directions” in Empathy in health professions education and patient care. (Heidelberg, New York, Dordrecht, London: Springer, Cham), 255–274.

[ref22] Hojat M. Axelrod D. Spandorfer J. Mangione S. (2013). Enhancing and sustaining empathy in medical students. Med. Teach. 35, 996–1001. doi: 10.3109/0142159X.2013.802300, PMID: 23758178

[ref23] Hojat M. DeSantis J. Shannon S. C. Mortensen L. H. Speicher M. R. Bragan L.. (2018). The Jefferson scale of empathy: a nationwide study of measurement properties, underlying components, latent variable structure, and national norms in medical students. Adv. Health Sci. Educ. 23, 899–920. doi: 10.1007/s10459-018-9839-9 PMC6245107 29968006

[ref24] Holden J.D. (2017). A toolkit to support nurse-patient communication through nurse-expressed empathy. doctoral dissertation. Minneapolis, MN: Walden University.

[ref25] Jeffrey D. (2016). Empathy, sympathy and compassion in healthcare: is there a problem? Is there a difference? Does it matter? J. R. Soc. Med. 109, 446–452. doi: 10.1177/0141076816680120, PMID: 27923897 PMC5154411

[ref26] Kataoka H. Iwase T. Ogawa H. Mahmood S. Sato M. DeSantis J.. (2019). Can communication skills training improve empathy? A six-year longitudinal study of medical students in Japan. Med. Teach. 41, 195–200. doi: 10.1080/0142159X.2018.1460657, PMID: 29683011

[ref27] Kerem E. Fishman N. Josselson R. (2001). The experience of empathy in everyday relationships: cognitive and affective elements. J. Soc. Pers. Relat. 18, 709–729. doi: 10.1177/0265407501185008, PMID: 35111922

[ref28] Kommalage M. (2011). Using videos to introduce clinical material: effects on empathy. Med. Educ. 45, 514–515. doi: 10.1111/j.1365-2923.2011.03951.x, PMID: 21486336

[ref29] Kourakos M. Vlachou E.D. Kelesi M.N. (2018). Empathy in the health professions: an ally in the care of patients with chronic diseases. Asklepieio Voulas: Institutional Repository Health Sciences.

[ref30] Lamm C. Batson C. D. Decety J. (2007). The neural substrate of human empathy: effects of perspective-taking and cognitive appraisal. J. Cogn. Neurosci. 19, 42–58. doi: 10.1162/jocn.2007.19.1.42, PMID: 17214562

[ref31] Lor K. B. Truong J. T. Ip E. J. Barnett M. J. (2015). A randomized prospective study on outcomes of an empathy intervention among second-year student pharmacists. Am. J. Pharm. Educ. 79:18. doi: 10.5688/ajpe79218, PMID: 25861099 PMC4386739

[ref32] Motataianu I. T. (2014). The empathy and communication—pride Personality's dimensions of the teacher. Procedia. Soc. Behav. Sci. 142, 708–711. doi: 10.1016/j.sbspro.2014.07.602

[ref33] Park K. H. Roh H. Suh D. H. Hojat M. (2015). Empathy in Korean medical students: findings from a nationwide survey. Med. Teach. 37, 943–948. doi: 10.3109/0142159X.2014.956058, PMID: 25182523

[ref34] Riess D. L. (2018). Effects of simulated clinical experiences on empathy, self-confidence, and satisfaction in nursing students. Minneapolis, MN: Walden University.

[ref35] Rohani C. Sedaghati Kesbakhi M. Mohtashami J. (2018). Clinical empathy with cancer patients: a content analysis of oncology nurses' perception. Patient Prefer. Adherence 12, 1089–1098. doi: 10.2147/PPA.S156441 29950822 PMC6016590

[ref36] Shin S. Park J.-H. Kim J.-H. (2015). Effectiveness of patient simulation in nursing education: meta-analysis. Nurse Educ. Today 35, 176–182. doi: 10.1016/j.nedt.2014.09.009, PMID: 25459172

[ref37] Sinclair S. Beamer K. Hack T. F. McClement S. Raffin Bouchal S. Chochinov H. M.. (2017). Sympathy, empathy, and compassion: a grounded theory study of palliative care patients' understandings, experiences, and preferences. Palliat. Med. 31, 437–447. doi: 10.1177/0269216316663499, PMID: 27535319 PMC5405806

[ref38] Singer T. Klimecki O. M. (2014). Empathy and compassion. Curr. Biol. 24, R875–R878. doi: 10.1016/j.cub.2014.06.054, PMID: 25247366

[ref39] Terezam R. Reis-Queiroz J. Hoga L. (2017). The importance of empathy in health and nursing care. Rev. Bras. Enferm. 70, 669–670. doi: 10.1590/0034-7167-2016-0032, PMID: 36988460

[ref40] Undung Y. De Guzman A. B. (2009). Understanding the elements of empathy as a component of care-driven leadership. J. Leadersh. Stud. 3, 19–28. doi: 10.1002/jls.20092

[ref41] Ward J. Cody J. Schaal M. Hojat M. (2012). The empathy enigma: an empirical study of decline in empathy among undergraduate nursing students. J. Prof. Nurs. 28, 34–40. doi: 10.1016/j.profnurs.2011.10.007, PMID: 22261603

[ref42] Williams B. (2014). A toolkit for educators and facilitators. Elsevier. 23–31. Available at: https://ltr.edu.au/resources/ID11_2059_Williams_Toolkit_2014.pdf.

[ref43] Williams B. Boyle M. Brightwell R. Devenish S. Hartley P. McCall M.. (2012a). An assessment of undergraduate paramedic students' empathy levels. Int. J. Med. Educ. 3, 98–102. doi: 10.5116/ijme.4fba.9190

[ref44] Williams B. Boyle M. Brightwell R. Devenish S. Hartley P. McCall M.. (2012b). Paramedic empathy levels: results from seven Australian universities. Int. J. Emerg. Serv. 1, 111–121. doi: 10.1108/20470891211275902

[ref45] Williams B. Boyle M. Earl T. (2013). Measurement of empathy levels in undergraduate paramedic students. Prehosp. Disaster Med. 28, 145–149. doi: 10.1017/S1049023X1300006X, PMID: 23351192

[ref46] Williams B. Boyle M. Howard S. (2015a). Empathy levels in undergraduate paramedic students. Int. J. Caring Sci. 8, 59–68. doi: 10.1007/s10459-018-9839-9

[ref47] Williams B. Brown T. McKenna L. Boyle M. Palermo C. Molloy E.. (2014a). Can DVD simulations be used to promote empathic behaviours and interprofessional collaboration among undergraduate healthcare students. Sydney, Australia: Monash University Press.

[ref48] Williams B. Brown T. McKenna L. Boyle M. J. Palermo C. Nestel D.. (2014b). Empathy levels among health professional students: a cross-sectional study at two universities in Australia. Adv. Med. Educ. Pract. 5:107. doi: 10.2147/AMEP.S57569 24833947 PMC4014368

[ref49] Williams B. Brown T. McKenna L. Palermo C. Morgan P. Nestel D.. (2015b). Student empathy levels across 12 medical and health professions: an interventional study. J. Compassionate Health Care 2, 4, 1–6. doi: 10.1186/s40639-015-0013-4

[ref50] Yang C.-P. P. Hargreaves W. A. Bostrom A. (2014). Association of empathy of nursing staff with reduction of seclusion and restraint in psychiatric inpatient care. Psychiatr. Serv. 65, 251–254. doi: 10.1176/appi.ps.201200531, PMID: 24492902

