# Supplementary material for: Effect of empathy training on the empathy level of healthcare providers in Ethiopia: a cluster randomized controlled trial
Source: Front Psychol. 2023 May 22;14:1091605. doi: 10.3389/fpsyg.2023.1091605 (PMC10239930; doi:10.3389/fpsyg.2023.1091605)
Supplement: Supplementary file 1 [file Table_1.pdf]

Table 1. Detail descriptions of Intervention protocol of empathy training

S.No	Intervention Packages	Components	Materials required	Skills to be enhanced	Delivery mechanisms	Duration of delivery
1	**Pretest survey**	Cognitive competency	JSE tool		Self-administered	10 minutes
**2**	Empathy video and PPT	Cognitive competency	Laptop<br>LCD	Cognitive empathy	Video show and PPT presentation	One hour
**3**	Empathy matching cards	Cognitive & communication competency	Pen<br>Notepad<br>Marker<br>Flipchart<br>Table	Teamwork spirit communication, & self-reflection	Participants were made to match each term with its definition in groups from randomly placed cards, group problem solving through discussion, & reflection	One hour
**4**	Storytelling and role-playing	Perspective taking	Pen<br>Flipchart<br>Marker<br>Notepad	Cognitive empathy, Communication, listening, self-reflection (expand understanding of new perspectives), & networking	Ask participants to share their past experiences while caring for women with obstetric fistula (upsetting/gladding, and things happened to them, how they read non-verbal cues, & decisions made), ask volunteers to role-play as client and care providers, and group reflection & discussion	One hour
**5**	Virtual patient & empathy toy videos	Understanding feelings and communicating those feelings	Laptop<br>LCD<br>Pen<br>Flipchart<br>Marker<br>Notepad	Affective and cognitive empathy, listening, self-reflection, & use of multisensory	Video show on empathic communication & empathy toy<br>Making participants take notes and reflect back then after	One hour

**6**	"If I was the patient activity":	Standing in patients' shoes	Flipchart Marker	Affective empathy, listening, & self-reflection	After the video show participants were asked to respond with what they felt if they were the patient in the video (identify missteps & compromised values as a care provider)	20minutes
**7**	Wrap up video on critical steps of ways how to improve empathy	Intention to help and provide a dignified care	Flipchart Marker	Self-reflection Perceived empathy	Asking for reflection on what they learned and their future application of empathy	30minutes
**8**	Post-intervention survey at one week, after a month, and three months.	Cognitive and affective Competency	JSE tool	Cognitive and affective empathy	Self-administered	10minutes each
