# Supplementary material for: Effect of empathy training on the empathy level of healthcare providers in Ethiopia: a cluster randomized controlled trial
Source: Front Psychol. 2023 May 22;14:1091605. doi: 10.3389/fpsyg.2023.1091605 (PMC10239930; doi:10.3389/fpsyg.2023.1091605)
Supplement: Supplementary file 2 [file Table_2.pdf]

Table 2. Empathy matching cards (Source Macquarie dictionary)

S.N	Empathy Matching Cards	Definitions
**1.**	Empathy	Entering into the feeling or spirit of a person or thing; appreciative perception or understanding.
**2.**	Sympathy	The fact or the power of entering into the feelings of another, especially in sorrow or trouble; fellow feeling, compassion, or commiseration.
**3.**	Compassion	A feeling of sorrow or pity for the sufferings or misfortunes of another.
**4.**	Caring	Exchanges of confidence, particularly in relation to some distressing experiences, which are intended to promote emotional healing.
**5.**	Emotion	Any of the feelings of joy, sorrow, fear, hate, love, etc.
**6.**	Rapport	Connection, especially harmonious or sympathetic relation.
**7.**	Affinity	A natural liking for, or attraction to, a person or thing.
**8.**	Pity	Sympathetic or kindly sorrow excited by the suffering or misfortune of another, often leading one to give relief or aid or to show mercy.
